# A case of spontaneous acalculous gallbladder perforation difficult to diagnose on computed tomography

**DOI:** 10.1093/jscr/rjag824

**Published:** 2026-09-22

**Authors:** Manabu Sato, Kazuki Hayashi, Akihiro Kobayashi, Koichiro Kubo, Nobuyoshi Yamazaki, Ryosuke Kobayashi, Fumitake Suzuki, Daichi Asai, Yuta Sannomiya, Naohiro Tomita, Noriko Yamada, Tomohiro Watanabe, Masaki Kakimoto, Kei Hasegawa, Kenji Ogata

**Affiliations:** Department of Surgery, Chiba Nishi General Hospital, 107-1, Kanegasaku, Matsudo 270-2251, Chiba, Japan; Department of Surgery, Chiba Nishi General Hospital, 107-1, Kanegasaku, Matsudo 270-2251, Chiba, Japan; Department of Surgery, Chiba Nishi General Hospital, 107-1, Kanegasaku, Matsudo 270-2251, Chiba, Japan; Department of Surgery, Chiba Nishi General Hospital, 107-1, Kanegasaku, Matsudo 270-2251, Chiba, Japan; Department of Surgery, Chiba Nishi General Hospital, 107-1, Kanegasaku, Matsudo 270-2251, Chiba, Japan; Department of Surgery, Chiba Nishi General Hospital, 107-1, Kanegasaku, Matsudo 270-2251, Chiba, Japan; Department of Surgery, Chiba Nishi General Hospital, 107-1, Kanegasaku, Matsudo 270-2251, Chiba, Japan; Department of Surgery, Chiba Nishi General Hospital, 107-1, Kanegasaku, Matsudo 270-2251, Chiba, Japan; Department of Surgery, Chiba Nishi General Hospital, 107-1, Kanegasaku, Matsudo 270-2251, Chiba, Japan; Department of Surgery, Chiba Nishi General Hospital, 107-1, Kanegasaku, Matsudo 270-2251, Chiba, Japan; Department of Surgery, Chiba Nishi General Hospital, 107-1, Kanegasaku, Matsudo 270-2251, Chiba, Japan; Department of Surgery, Chiba Nishi General Hospital, 107-1, Kanegasaku, Matsudo 270-2251, Chiba, Japan; Department of Surgery, Chiba Nishi General Hospital, 107-1, Kanegasaku, Matsudo 270-2251, Chiba, Japan; Department of Surgery, Chiba Nishi General Hospital, 107-1, Kanegasaku, Matsudo 270-2251, Chiba, Japan; Department of Surgery, Chiba Nishi General Hospital, 107-1, Kanegasaku, Matsudo 270-2251, Chiba, Japan

**Keywords:** gallbladder perforation, computed tomography, emergency diagnostic laparoscopy

## Abstract

Gallbladder perforation is a rare and life-threatening event. Accurately diagnosing gallbladder perforation without cholecystitis or gallstones is difficult. The patient was admitted with abdominal pain and was diagnosed with diffuse peritonitis the following day via computed tomography (CT). Laparoscopic findings revealed the accumulation of bile-like ascites and a thinned and soft gallbladder with slight leakage of bile juice from a pinhole perforation. Laparoscopic cholecystectomy and peritoneal lavage were then performed. In the present case, it was difficult to diagnose gallbladder perforation because of the lack of cholecystitis and gallstones in the CT findings. If preoperative CT shows an acute abdomen with peritoneal effusion, especially accumulated around the gallbladder, then gallbladder perforation may be considered as a primary lesion.

## Introduction

Gallbladder perforation is a rare event, and idiopathic perforation without any obvious causes, such as cholecystitis or cholecystolithiasis, is extremely rare [1, 2]. The reported mortality rate of gallbladder perforation is 12%–42% [2, 3]. Although gallbladder perforation is a serious condition, it is poorly reported, and the perioperative management of gallbladder perforation is often challenging. In the emergency surgery decision making process, abdominal computed tomography (CT) is important because it is specific and sensitive in showing gallbladder stones, air in its wall, and pericholecystic fluid around it [4]. In contrast, if abdominal CT shows the lack of obvious cholecystitis, a defect of the gallbladder wall, or cholecystolithiasis, then making a definite diagnosis is rather difficult.

In this report, we present a case of gallbladder perforation that reminds us that the diagnosis of gallbladder perforation without cholecystitis or gallstones is very difficult.

## Case report

**Figure 1:**
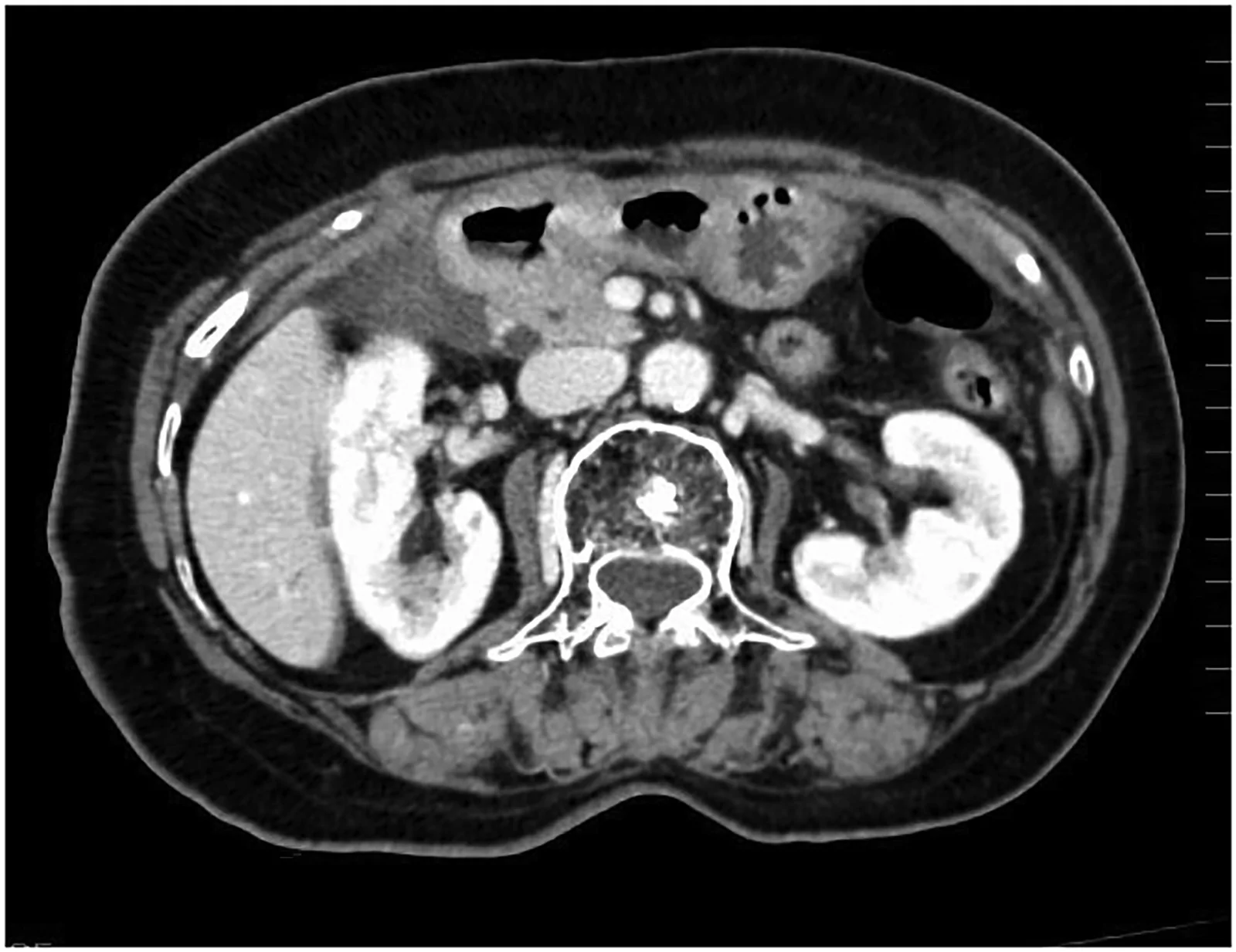
CT finding at admission showed mild gallbladder wall thickening, slight effusion around the gallbladder, and a lack of ascites.

**Figure 2:**
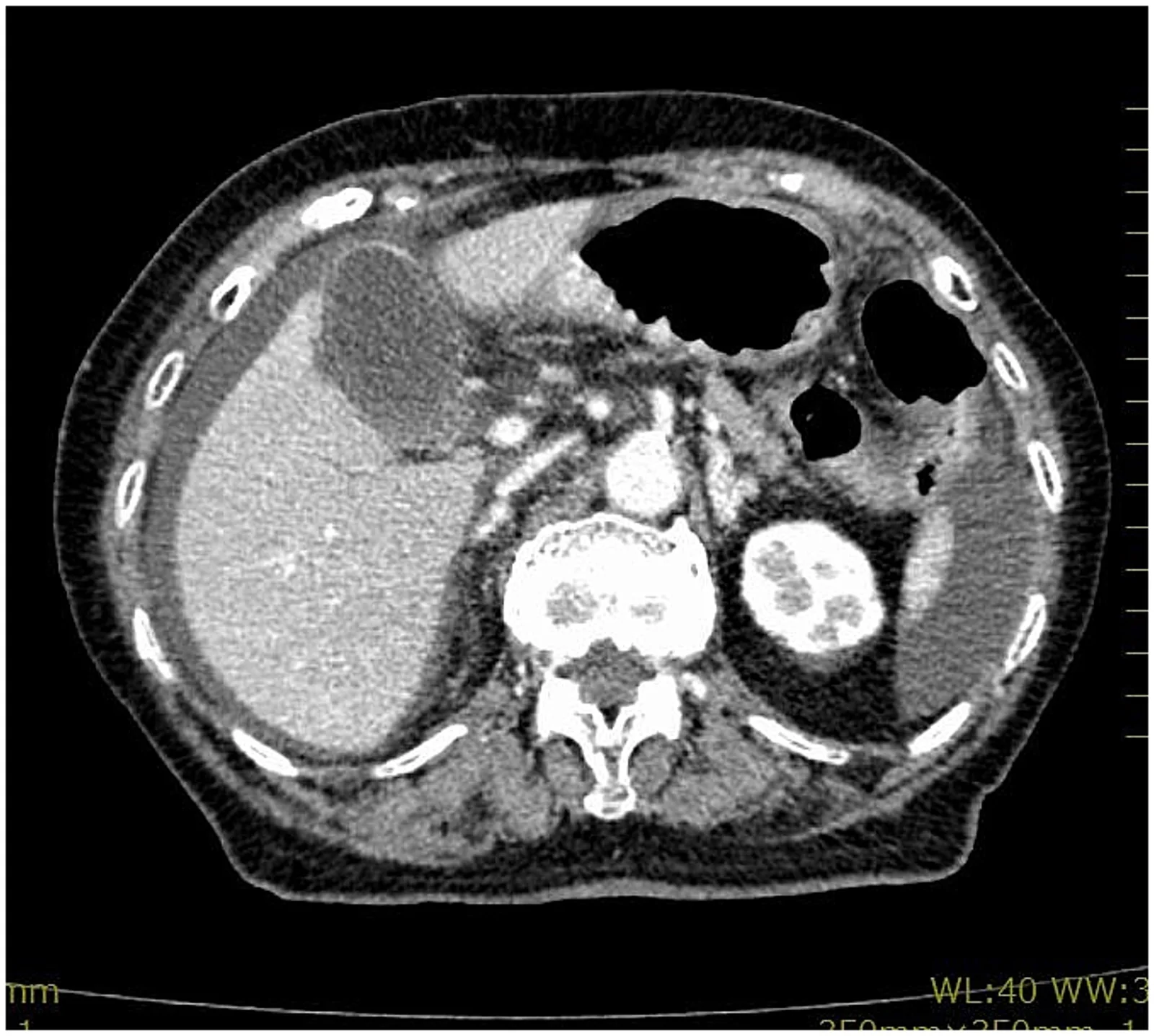
CT finding at the day after the admission showed increased ascites around the liver and paracolic gutter. The wall of a gallbladder was slightly thickened and edematous compared with yesterday, but the difference was slight.

**Figure 3:**
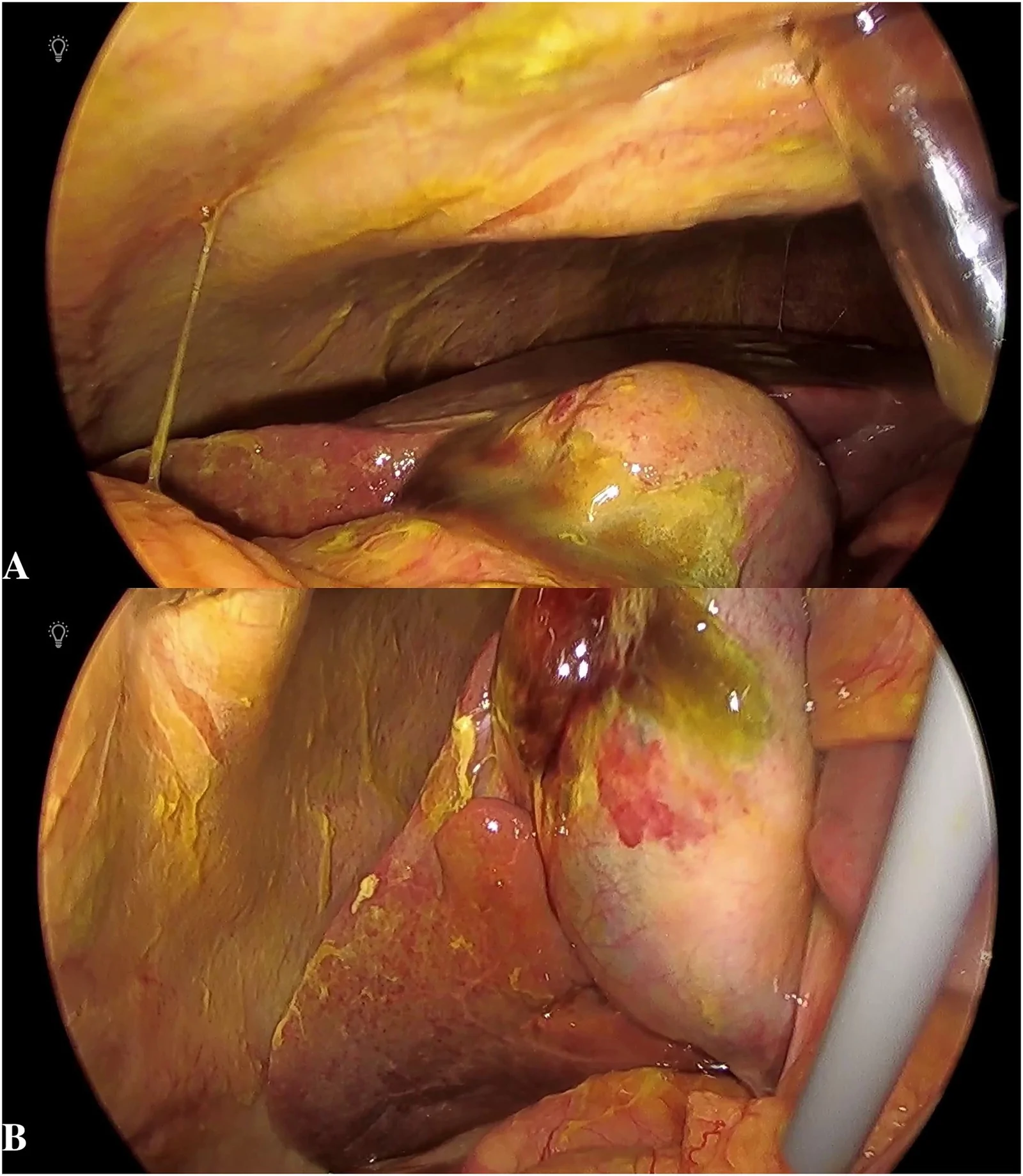
Laparoscopy findings. Laparoscopy showed the accumulation of bile-like ascites and a thinned and soft gallbladder with a slight leakage of bile juice from a pinhole perforation.

**Figure 4:**
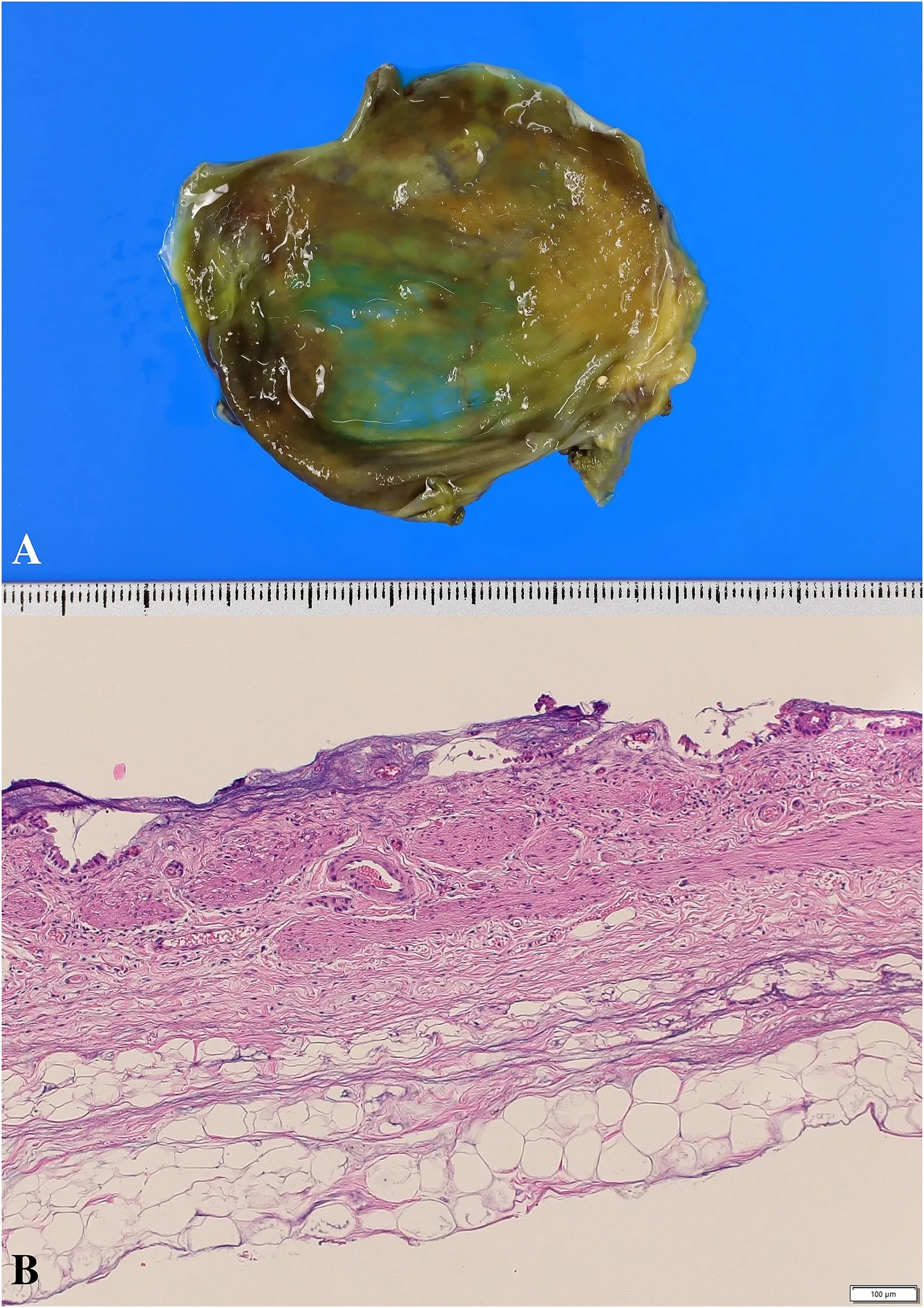
Macroscopic and microscopic findings. Macroscopic findings showed wall thickness of the gallbladder. Microscopic finding showed degeneration and necrotic change of the gallbladder wall. Slight neutrophilic infiltration also existed. (A) Macroscopic image. (B) Microscopic image.

An 85-year-old woman presented with a 1-day history of continuing abdominal pain. Physical examination revealed tenderness and slight guarding over the whole abdomen. The patient had no episodes of severe abdominal pain. Her medical history was hypertension, and previous surgical history was appendectomy for acute appendicitis. The laboratory findings at admission showed a normal WBC, C-reactive protein (CRP), and hepatobiliary enzymes. The findings of CT were mild gallbladder wall thickening, slight effusion around the gallbladder, and a lack of ascites (Fig. 1). The following day, the abdominal pain was worsening and her physical abdominal findings showed severe tenderness, rebound tenderness, and guarding. Re-examinations revealed slight increases of the WBC, CRP, serum amylase, without increases of hepatobiliary enzymes in a blood test. CT showed the appearance of ascites around the liver and paracolic gutter, but no marked wall thickness or inflammation of the gallbladder was observed. No other causes of peritonitis were detected (Fig. 2). Even though no clear diagnosis had been made, emergency diagnostic laparoscopy was performed because of the signs of diffuse peritonitis. The laparoscopic findings showed the accumulation of bile-like ascites and a thinned and soft gallbladder with a slight leakage of bile juice from a pinhole perforation (Fig. 3). No gallbladder volvulus was observed. Laparoscopic cholecystectomy and peritoneal lavage were performed after the diagnostic laparotomy. The pathological findings of the removed gallbladder revealed ischemic change and slight inflammation of the wall, without any neoplastic lesions (Fig. 4). Retrospective scrutinization after surgery of the CT findings indicated a perforation of the gallbladder.

## Discussion

Infections, malignancy, trauma, drugs (e.g. corticosteroids), and systemic diseases such as diabetes mellitus and atherosclerotic heart disease are common predisposing factors [3, 5]. Niemeirer *et al*. reported a classification system for gallbladder perforation in his study [6]. He categorized gallbladder perforation into three types: type 1, chronic perforations with the presence of a fistulous communication between the gallbladder and some other viscus; type 2, subacute perforations in which the perforated gallbladder is surrounded by an abscess walled off by adhesions from the general peritoneal cavity; and type 3, acute perforation of the gallbladder into the free peritoneal cavity without any protective adhesions.

Systematic reviews have shown that the proportions of type 1 and type 2 were ~30%–40%, 40%–45%, and type 3 was 10%–25%, and perforation was associated with cholelithiasis in 86% of the cases [2, 3]. Therefore, type 3 gallbladder perforation without cholelithiasis or cholecystitis is uncommon.

CT is a widely used imaging modality for gallbladder disease, including gallbladder perforation. However, gallbladder perforation without gallstones or cholecystitis via CT remains challenging. Patel *et al*. reported eight cases of gallbladder perforation in which CT examinations successfully confirmed the diagnosis [1].

Conversely, case reports of spontaneous gallbladder perforation without gallstones or cholecystitis have highlighted the difficulty of making a preoperative diagnosis [7, 8] no preoperative signs of biliary disease were observed, and the diagnosis was confirmed laparoscopically upon detecting bile peritonitis caused by leakage from the gallbladder wall.

The perforation was missed here due to multiple factors—no stones, minimal inflammation, normal liver tests, minimal wall thickening. When diffuse biliary peritonitis is suspected but imaging is non-diagnostic, diagnostic laparoscopy should not be delayed.

## Conclusion

We encountered a case of gallbladder perforation that was difficult to diagnose preoperatively due to the absence of gallstones or cholecystitis on CT. Gallbladder perforation is a rare and life-threatening condition. We acknowledge the importance of considering gallbladder perforation in the differential diagnosis when diffuse peritonitis with perineal effusion around the gallbladder is observed.
